# An Overview of Systemic Targeted Therapy in Renal Cell Carcinoma, with a Focus on Metastatic Renal Cell Carcinoma and Brain Metastases

**DOI:** 10.3390/cimb45090485

**Published:** 2023-09-21

**Authors:** Liliana Eleonora Semenescu, Amira Kamel, Vasile Ciubotaru, Silvia Mara Baez-Rodriguez, Mircea Furtos, Alexandra Costachi, Anica Dricu, Ligia Gabriela Tătăranu

**Affiliations:** 1 Department of Biochemistry, Faculty of Medicine, University of Medicine and Pharmacy of Craiova, Str. Petru Rares nr. 2-4, 710204 Craiova, Romania; lilisicoe@yahoo.com (L.E.S.); alexandra.costachi@yahoo.com (A.C.); 2 Neurosurgical Department, Clinical Emergency Hospital “Bagdasar-Arseni”, Soseaua Berceni 12, 041915 Bucharest, Romania; kamel.amyra@yahoo.com (A.K.); dr_vghciubotaru@yahoo.com (V.C.); mara.silvia@icloud.com (S.M.B.-R.); ligia.tataranu@umfcd.ro (L.G.T.); 3 Neurosurgical Department, University Emergency Hospital of Bucharest, 050098 Bucharest, Romania; mirceaf90@yahoo.com; 4 Department of Neurosurgery, Faculty of Medicine, University of Medicine and Pharmacy “Carol Davila”, 020022 Bucharest, Romania

**Keywords:** brain metastases, renal cell carcinoma, systemic therapy, immune checkpoint inhibitors, immunotherapy, neuro-oncology, targeted therapy

## Abstract

The most commonly diagnosed malignancy of the urinary system is represented by renal cell carcinoma. Various subvariants of RCC were described, with a clear-cell type prevailing in about 85% of all RCC tumors. Patients with metastases from renal cell carcinoma did not have many effective therapies until the end of the 1980s, as long as hormonal therapy and chemotherapy were the only options available. The outcomes were unsatisfactory due to the poor effectiveness of the available therapeutic options, but then interferon-alpha and interleukin-2 showed treatment effectiveness, providing benefits but only for less than half of the patients. However, it was not until 2004 that targeted therapies emerged, prolonging the survival rate. Currently, new technologies and strategies are being developed to improve the actual efficacy of available treatments and their prognostic aspects. This article summarizes the mechanisms of action, importance, benefits, adverse events of special interest, and efficacy of immunotherapy in metastatic renal cell carcinoma, with a focus on brain metastases.

## 1. Introduction 

Renal cell carcinoma (RCC) is a malignant tumor originating from the renal tubular epithelium, accounting for 80–90% of malignant renal tumors [1]. There are many tumor subtypes, and the multimodal therapeutic approach, especially immunotherapy, has exceedingly evolved in the last decades, dramatically transforming the natural evolution of the disease. In Europe and North America, the lifetime risk of developing RCC ranges between 1.3% and 1.8% [2,3]. Metastatic renal cell carcinoma (mRCC) has been reported to develop in almost 30% of patients with RCC, while brain metastases from renal cell carcinoma (BM RCC) have a reported incidence reaching 13% [4]. The occurrence of BM usually indicates a poor prognosis, with a low overall survival (OS) rate. The most common histological type of RCC that metastasizes in the brain is clear-cell renal cell carcinoma (ccRCC) [5]. From the initial palliative intents to new systemic therapies, profound changes have been made in this field, with promising results [6], such as the achievement of long-term survival and improvement of quality of life [7]. Furthermore, regarding multimodal approaches and targeted agents, researchers concluded that immunotherapy responses are enhanced by local radiotherapy [8], by modifying the blood–brain barrier (BBB) and permitting the access of immune cells [9], but also by releasing cancer antigens after cellular lysis, by surging presentation to immune cells, along with hindering immune-suppressing cells, inflammatory cytokine production, and the set-off of a particular anti-tumor response [7,10]. In the current era, new therapeutic options for solid tumors and metastasis are emerging and becoming accessible, counting tyrosine kinase inhibitors (TKIs), mostly vascular endothelial growth factor (VEGF) receptor inhibitors, and immune checkpoint inhibitors (ICIs) as lymphocyte antigen-4 (CTLA4) and anti-programmed death 1 (PD-1) [7,11,12,13]. An important association between various molecular alterations and RCC subtypes has been established.

In ccRCC, a recurring alteration of the von Hippel–Lindau (*VHL*) gene has been described as leading to angiogenesis through the transcription of genes managed by hypoxia-inducible factor (HIF) like VEGF [14,15,16]. In type 1 papillary RCC (pRCC), alterations in the mesenchymal epithelial transition (MET) TK receptor are more frequent in comparison to type 2 pRCC, which is less common [17]. In addition, type 2 pRCC is more often associated with mutations in set domain containing 2 (*SETD2), cyclin-dependent* kinase inhibitor 2A (*CDKNA2A*), moesin-ezrin-radixin-like (MERLIN) tumor suppressor (*NF2*), and telomerase reverse transcriptase (*TERT*) [17].

Notwithstanding the fact that chromophobe RCC (chRCC) very seldom metastasizes, mitochondrial alterations, tumor protein p53 (*p53*) mutations, and activation of mammalian target of rapamycin (mTOR) pathways have been described [15]. 

Among familial RCC syndromes, the fumarate hydratase (*FH*) germline mutation that interferes with the function of the Krebs cycle has been highlighted [18], causing hereditary leiomyomatosis and renal cell cancer (HLRCC) and being associated with an aggressive form of papillary RCC (pRCC) [19]. No specific systemic therapy has been established for this pathology, but targeting VEGF and EGFR is an option [19]. The mTOR inhibition did not show notable benefits in HLRCC [20]. Another hereditary RCC syndrome includes von Hippel–Lindau disease, associated with a risk of developing ccRCC, caused by *VHL* inactivation and activation of hypoxia-inducible factor 2α (HIF-2 α) [21,22]. HIF-2 α inhibitors have been studied in this pathology, with encouraging results [21]. Birt–Hogg–Dubé (BHD) syndrome is associated with RCC in 25% of individuals, with ccRCC, chromophobe RCC, and pRCC cases described [23,24]. However, metastatic disease in these cases is rare [25]. Hereditary papillary RCC is correlated with genetic alteration in *MET*, and hereditary type 1 pRCC [26]. Due to the MET genetic alteration, patients with metastatic hereditary pRCC could consider MET inhibitor treatment [27]. Succinate dehydrogenase-related RCC is associated with a lifetime risk for RCC of less than 15% [22]. Hereditary BAP1-associated RCC, constitutional chromosome 3 translocations, and familial non-syndromic RCC have been described in the category of hereditary RCC syndromes [22].

The relationship between many of the involved aforementioned genes, factors, and pathways and mRCC and BM RCC will be discussed further.

## 2. Clear-Cell Renal Cell Carcinoma

Clear-cell RCC (ccRCC) is the most common histological type of RCC [28], accounting for approximately 60% of all cases and 75–80% of metastatic cases, and is considered both an angiogenic and immunogenic tumor. ccRCC occurs in 50–70% of the cases due to mutations in the *VHL* gene [1,29,30]. A correlation between the function loss of VHL and the stabilization of HIFα has been stated [31]. Although therapeutic options available for advanced stages of ccRCC are limited, great efforts are made in order to uncover new treatment alternatives. 

The incidence of BM in individuals with ccRCC has been estimated to be 8% [3], with described cases of leptomeningeal metastases [32]. Metastatic ccRCC exhibits an increased expression of MET, especially in BM from ccRCC, and this is particularly important when it comes to systemic agents as treatment modality for BM RCC [33]. Lately, cabozantinib, which is a MET inhibitor, has shown potential efficacy in BM from ccRCC [34,35].

## 3. Non-Clear-Cell Renal Cell Carcinoma

Non-clear-cell renal cell carcinomas (nccRCC) account for nearly 25% of all kidney cancers and include a group of highly heterogeneous tumors concerning histological varieties and oncogenic abnormalities [36,37]. A significant number of studies assessing the efficacy of immunotherapy in this category of patients have been conducted, and they will be discussed further. The most frequently encountered subtypes of nccRCC comprise the papillary subtype (~15%), chromophobe RCC (up to 7%), collecting duct RCC (~2%), renal medullary carcinoma (<1%), and translocation carcinomas (<1%) [38].

tRCC is a rare subtype of RCC that is relatively poorly described. In tRCC, a microphthalmia/transcription factor E (*MiT/TFE*) gene fusion has been described. The outcomes of patients with tRCC treated with VEGFR-TKI are worse than those treated with ICIs (median OS 62.4 months with ICI and median OS 10.3 months with TKI) [39]. Since sarcomatoid characteristics can be found in any other histologic subtype of RCC, sarcomatoid RCC is not viewed as a different subtype [40]. In localized disease stages, the main therapeutic approach is similar to that of the ccRCC, whereas in the metastatic stages, immunotherapy has been proven to be effective. Furthermore, in metastatic sarcomatoid RCC, it has been stated that patients exhibit positive responses to ICI therapy [40,41,42].

The incidence of brain involvement in nccRCC has been estimated to be 3% in pRCC and 2% in chRCC [3].

To date, we have no approved therapy specifically indicated for metastatic nccRCC [43]. It has been reported that patients with chRCC do not benefit from immunotherapy, while individuals with pRCC show a therapeutic response of approximately 35% [44]. However, several studies regarding immunotherapy in patients with nccRCC proved significant benefits of immunotherapy in this category of individuals.

Alterations of MET, TERT, and EGFR have also been described in papillary RCC (pRCC), especially in type 1, while type 2 is more frequently defined by alterations in *CDKN2A*, *SETD2*, *TFE3*, and the nuclear factor E2-related factor 2 (NRF2)—antioxidant response element (ARE) pathway [45,46,47]. Furthermore, in type 2 pRCC, a CpG island methylator phenotype (CIMP) was noticed, and it was described by genetic alteration of the FH encoding gene and associated with a poor prognosis [45,46]. These results are precisely significant in pRCC, as they may encourage further studies regarding systemic agents with MET inhibitory features, such as crizotinib, cabozantinib, or savolitinib [48,49,50].

## 4. Brain Metastases from RCC

Metastatic renal cell carcinoma (mRCC) occurs in approximately 30% of patients with RCC, and patients who, at the initial diagnosis, have BM usually exhibit a poor prognosis. According to some authors, untreated patients with BM RCC may have a median overall survival (OS) of at least 4 months [4,11]. According to the latest evidence, BM RCC is primarily managed with neurosurgery and/or radiotherapy (RT) [51,52,53,54], with additive therapeutic benefits from systemic therapy, such as immunotherapeutic agents or anti-angiogenic factors [55]. It has been stated that, with very few exceptions [56], frontline therapy using VEGFR TKI or mTOR inhibitors in monotherapy is no longer in use, as currently the main options are represented by combinations of various systemic agents, especially ICIs [56,57]. Infrequently, the eradication of the primary RCC can trigger an immunological response that will result in spontaneous remission in metastases [58]. Some studies suggest that patients who require immunotherapy in first-line settings should be stratified according to the presumed risk into International mRCC Database Consortium risk groups (respectively, 0—favorable, 1–2—intermediate, 3—poor), stating that patients with poor risk or intermediate risk would be better off being offered a combination of two ICIs or an ICI associated with a VEGFR TKI [59]. In comparison with a subsequent administration, patients treated beforehand with immunotherapy had a better OS rate without reports of significant toxicity after the treatment with the most frequent ICIs (nivolumab, pembrolizumab, atezolizumab, and durvalumab) [60]. Notwithstanding the fact that the therapeutic approach of RCC has drastically changed with the arrival of ICIs, no significant effectiveness of this category of targeted agents was stated specifically in patients with BM RCC [4,11], partly because antitumor benefits of ICIs could be cancer-specific [11].

BM grows as a result of a cellular tumor spreading throughout the blood into the brain vasculature [61]. However, seeding from an existing metastasis is not excluded [61]. The development of a new metastasis (colonization) is a result of intricate microenvironmental interactions, neuroinflammatory cascades, and the formation of new blood vessels [61]. Neovascularization is a process directly managed and influenced by an equilibrium among anti-angiogenic factors and pro-angiogenic factors [62]. Essential elements with pro-angiogenic features are represented by vascular endothelial growth factors (VEGFs) and platelet-derived growth factors (PDGFs) [62]. These growth factors bind to receptors available on the cell surface, called receptor tyrosine kinases (TK), and this process will activate the TK [62,63]. Although the increasing action of angiogenesis is a consequence of overexpression or mutation of growth factors and receptor TK, tumoral development can also be influenced by ischemic circumstances when the available vascularization is not able to supply enough oxygen [62,64]. As a reaction to the lack of oxygen, HIF will start to induce gene transcription for neovascularization and tumoral development factors [62,63,64]. Despite the fact that the VHL gene typically regulates HIF, in RCC, the altered function of this gene leads to increased production of growth factors and tumors by activating hypoxia pathways [62,64]. In addition, activation of the mammalian target of rapamycin (mTOR) represents another pathway for increasing the expression of HIF [65].

A rising number of systemic therapies are being developed for mRCC, including immune checkpoint inhibitors (ICIs), which have also been studied in patients with BM RCC [11]. Even though patients with metastatic ccRCC may benefit from ICI therapies [66], those specific patients with BM RCC may not benefit as much from ICI treatment [11], also given the modest penetrance of the BBB [67]. However, in a multimodal approach, when combined with stereotactic radiosurgery and stereotactic radiotherapy, ICI can decrease the occurrence of new BM and even prolong survival [68,69,70,71].

A high density of CD8+ T cells in ccRCC is correlated with a poor prognosis and may suggest that immune invasion is of great importance in this pathology [72,73]. It has also been suggested that in BM from ccRCC, the response to ICI is reduced by the brain microenvironment and that the BM stage is an indication of the ability of tumoral cells to avoid the immune system [74].

PI3K and mTOR inhibitors showed satisfactory penetration of the BBB, in preclinical studies, suggesting a new possible therapeutic interest for BM [75]. In RCC pathology, the inactivation of VHL has been correlated to VEGF expression, and antibodies to VEGF have been studied (e.g., bevacizumab), with good results in individuals with mRCC [76].

## 5. Available Systemic Agents for Patients with mRCC and BM RCC

Targeted agents for mRCC and BM RCC are commonly given either as monotherapy or in combination. The major classes of systemic therapies are summarized in Table 1. 

The proposed systemic therapy for different types of mRCC, according to the latest major international guidelines, is summarized in Table 2.

## 6. Trial-Eligible versus Trial-Ineligible Patients

The trial eligibility and ineligibility of patients included have been defined according to the common exclusion criteria found in phase III clinical trials [133]. Immune-oncology-based therapies have been approved based on randomized clinical trials; however, these trials do not include a sizable portion of patients, and a large part of real-world patients with solid tumor malignancies are ineligible for current clinical trials, which results in significantly worse outcomes compared to trial-eligible patients [42].

Strict inclusion criteria for oncology clinical trials, of which worth mentioning are Karnofsky Performance Score ≥70% and/or Eastern Cooperative Oncology Group Performance Status of 0–1, absence of active central nervous system metastases, very often represent a major structural obstacle to patient enrollment [134,135]. Discrepancies in renal function assessment and the presence of cardiovascular disease are also counted among the barriers encountered by patients in these cases [135,136]. Regarding patients with BM RCC, eligibility criteria in RCC trials are even more problematic. Recent studies report that approximately 33–38% of RCC clinical trials excluded patients with BM [135,137], while 62% included these patients only if the BM were not active [135]. Furthermore, most studies exclude patients with BM RCC treated with prior glucocorticoids for brain edema, as this treatment will reduce the blood–brain barrier (BBB) permeability of systemic agents [79]

Consequently, accrual rates are low, and the results have poor generalizability. Strict criteria for clinical trial eligibility are for safety reasons and to maintain internal validity and patient homogeneity within the clinical trial, and 35% of mRCC patients would not have met the eligibility criteria for VEGF-targeted therapy clinical trials based on routine exclusion criteria [134]. Regarding generalizability, current eligibility criteria are considered unable to eliminate clinically notable dissimilarities between study populations and the real-world population [138]. As a solution, it has been suggested to conduct a thorough examination of the risk/advantage ratio, in addition to extensive inclusion criteria. The eligibility criteria for oncology clinical trials should be broadened [42,139]. 

## 7. The Abscopal Effect

The subject of the so-called abscopal effect has been of great interest in the oncological field, and it refers to an isolated phenomenon by which local radiation therapy (RT) instigates an immune-mediated anti-tumor effect at a distant site, inducing tumor shrinkage. The field is still of much interest, as many authors suggest good overall results after inducing the abscopal effect in patients with BM as a result of a combination of radiotherapy and immune checkpoint inhibitors [3,140]. This phenomenon can be used as a stimulator of the immune system against primary tumors in patients with BM RCC [141]. Regarding the mechanism of action, there is evidence suggesting that certain radiation doses can induce an optimal amount of DNA double-stranded breaks, initiating an anti-tumor response [142].

## 8. Systemic Agents in Monotherapy

It has been stated that cancer cells and tumor-infiltrating cells contribute to the process of drug resistance, or the lack/absence of a response to treatment strategies [143]. However, systemic therapy has been proven to be of great interest given its efficacy in RCC, either in monotherapy or in combination, as well as in the setting of a multimodal therapeutic approach. Although systemic agents are still a moving target, many studies have demonstrated significant benefits and promising results in different types of RCC and BM RCC. The NIVOREN Multicenter Phase II Study was the first prospective study that included BM RCC individuals, even though it did not conclude a significant benefit of nivolumab monotherapy administration in patients with previously untreated BM RCC [144]. Additionally, the study approaches the matter of radiotherapy in combination with immunotherapy and concludes a 6-month intracranial progression-free survival rate of 23.8% in individuals with untreated BM RCC versus 49.4% in those treated with prior local therapy [144]. Nivolumab in monotherapy administered before nephrectomy did not show beneficial effects in individuals with a high risk of remission, especially when compared with surgery alone [145]. McFarlane J. J. et al. approached the subject of the effectiveness of nivolumab monotherapy in patients with advanced ccRCC. The authors validate the benefits and safety of this therapeutic agent in the same dose as mentioned for the nccRCC patients, concluding a median survival rate of 21.8 months [146], in comparison to administration of everolimus (19.6 months) [147], nivolumab plus ipilimumab (18 months) [18], pembrolizumab (10.3 months), or chemotherapy (7.4 months) [148,149]. Nivolumab in combination with ipilimumab demonstrated significant efficacy in previously untreated mRCC patients, especially in terms of OS, objective response rate (ORR), and PFS [18]. The 12-month OS was 80%, the 18-month OS was 75%, ORR 42% (in comparison with 27% in the sunitinib monotherapy group), and the median PFS was 11.6 months (in comparison with 8.4 months in the sunitinib monotherapy group), with an 81% response of at least 1 year in intermediate/poor risk individuals [18]. 

The efficacy of systemic agents in monotherapy, like sunitinib or pazopanib, has been recently tested in patients with BM RCC in a 2023 study by Takemura et al. The authors evaluated 775 patients with BM RCC, assessing the efficacy of first-line immuno-oncology (IO) therapy in combination (Cohort 1) with sunitinib monotherapy or pazopanib monotherapy (anti-VEGF monotherapy, Cohort 2). The study concluded better results in patients with BM RCC in Cohort 1 when compared to Cohort 2. In Cohort 1, a complete response was achieved in 3.4%, a partial response was achieved in 25.9%, stable disease was described in 39.7%, and progressive disease in 31%. In Cohort 2, a complete response was achieved in 0.7%, a partial response was achieved in 29.6%, stable disease was described in 36.7%, and progressive disease in 33.0%. Factors like neurosurgical intervention, SRS, and IO therapy were correlated with longer survival rates [150]. The effectiveness of sunitinib or sorafenib in monotherapy versus placebo has been evaluated in patients with resected unfavorable RCC (ASSURE phase III trial), with non-elucidating results: 5-year OS of 75.2% for sunitinib, 80.2% for sorafenib, and 76.5% for placebo [151]. However, due to an unfavorable adverse effects profile and no survival advantage in sunitinib and sorafenib, none of these drugs are recommended in monotherapy [152]. KEYNOTE-564 study concluded a better disease-free survival in patients with ccRCC treated with pembrolizumab, as a post-nephrectomy adjuvant therapy compared with placebo. No deaths were attributed to pembrolizumab and the estimated number of individuals alive at 30 months was 95.7% with pembrolizumab and 91.4% with placebo [153]. Noticeably, atezolizumab did not prove efficacy in monotherapy in patients with mRCC (DFS of 57.2 months for atezolizumab versus 49.5 months for placebo, with no OS differences) [154].

**Figure 1:**
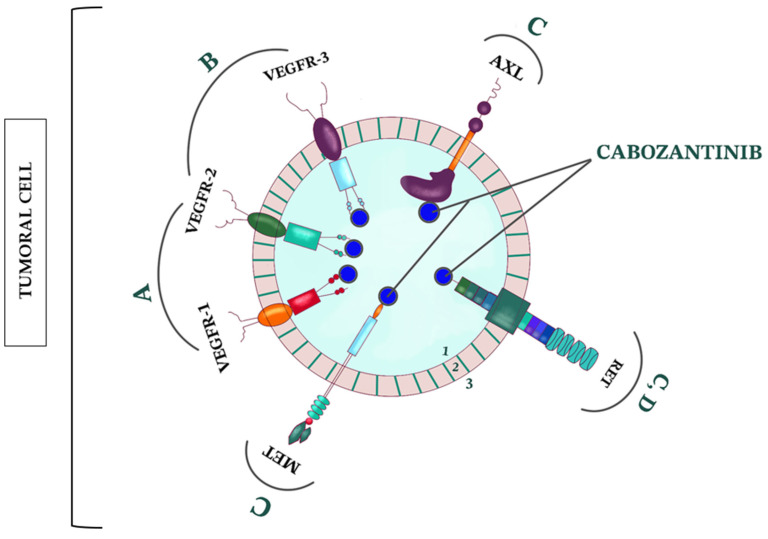
Mechanism of action of cabozantinib (also known as XL-184 or BMS-907351), a micromolecule that inhibits multiple receptor tyrosine kinases implicated in tumor growth, angiogenesis, drug resistance, and metastatic progression of cancer. VEGFR—vascular endothelial growth factor receptor; AXL—the gene AXL, located at chromosome 19q13.2 (originating from the Greek word “anexelekto”, translated as “uncontrolled”), is encoding a protein called AXL; RET—rearranged during transfection (a protooncogene responsible for encoding a receptor TK; this receptor plays a critical role in the formation of neural crest-derived cell lineages and genitourinary tract; MET—mesenchymal-epithelial transition factor (a type of receptor TK, usually revealed on the surface of several types of epithelial cells; 1—intracellular space; 2—cellular membrane; 3—extracellular space; A—angiogenesis; B—lymphangiogenesis; C—tumoral growth, metastatic invasion; D—indirectly stimulates the tumoral growth, invasion, and metastatic evolution; pro-inflammatory role in the tumor microenvironment.

Lately, cabozantinib demonstrated encouraging results in RCC pathology. When compared to the administration of everolimus, cabozantinib alone demonstrated superior efficacy and safety in treating patients with advanced RCC, in the METEOR trial. The median OS concluded by the study was 21.4 months in the cabozantinib group versus 16.5 months in the everolimus group [15]. Dysregulating the signaling of MET, AXL, and VEGFR can damper the anti-tumor immune response. Cabozantinib is the only approved multitargeted TKI that inhibits MET, AXL, and VEGFR [155,156,157,158,159,160,161,162,163,164,165,166]. Cabozantinib in monotherapy and combined with nivolumab showed important benefits in BM RCC cases that were resistant to RT and ICIs [86]. Furthermore, a macroscopic complete response of the BM RCC and perilesional edema reabsorption were observed after 12 weeks of second-line treatment with cabozantinib [167]. In like manner, cabozantinib in monotherapy demonstrated important benefits in patients with BM RCC, with a complete treatment response [34,85]. Given the fact that cabozantinib showed such promising results with a complete macroscopical intracranial response, we chose to depict its mechanism of action in Figure 1.

In BM RCC cases treated with cabozantinib monotherapy, median OS rates of 8.8 months have been achieved [35]. Cabozantinib has been proven to have great efficacy and safety regarding BM as well as extracranial activity (intracranial response rates of 55% and 47%; extracranial response rates of 48% and 38%; median OS of 15 and 16 months) [168]. Phase II Alliance A031203 CABOSUN trial showed a median PFS of 8.2 months in patients with ccRCC treated with cabozantinib and 5.6 months with sunitinib, while cabozantinib reduced the rate of disease progression or death by 34% compared with sunitinib [169,170]. When comparing post-nephrectomy everolimus administration to placebo, the EVEREST study (estimated study completion: 15.10.2026) concluded shorter recurrence-free survival rates in the latter group (64% versus 61%), but with similar OS rates [171]. Adjuvant therapy with girentuximab (cG250), a chimeric IgG antibody, did not show significant efficacy when compared to placebo in individuals with high-risk ccRCC [172]. Campbell et al. conducted a study regarding the administration of tremelimumab monotherapy or in combination with cryoablation in individuals with mRCC. The results favored the monotherapy, with a median OS of 33.7 months in comparison to 22.7 months for the combined treatment. Worth mentioning is the fact that, notwithstanding the therapeutical choice, ccRCC histology had better outcomes when compared to nccRCC cases [173].

Several studies approach the matter of systemic therapy as monotherapy in nccRCC patients. Lee et al. realized a non-randomized, open-label, multicenter prospective phase II study in Korean patients with metastatic nccRCC, concluding that sunitinib showed promising results in these patients (median progression-free survival was 6.4 months, and the 1-year progression-free survival rate was 40%, with a 1-year survival rate of 65%) [100]. The phase II KEYNOTE-427 study (cohort B) aims to evaluate the efficacy and safety of pembrolizumab in monotherapy in individuals with advanced nccRCC. The study concluded that patients with advanced nccRCC could benefit from first-line pembrolizumab in monotherapy (median progression-free survival was 4.2 months; the 24-month rate was 18.6%; median overall survival was 28.9 months, and the 24-month rate was 58.4%) [128].

When assessing the efficacy of crizotinib in individuals with pRCC type 1, of a total of four MET+ patients, two had a partial response, while one had stable disease (phase II clinical trial CREATE) [105]. Regarding the administration of savolitinib monotherapy in patients with metastatic pRCC, it has been stated that MET+ pRCC individuals had a longer progression-free survival in comparison to MET-independent disease (6.2 months versus 1.4 months) [174]. The efficacy of sunitinib, cabozantinib, crizotinib, or savolitinib in patients with metastatic papillary RCC has been tested in the phase II multicentric clinical trial PAPMET. The study concluded a median progression-free survival of 5.6 months for sunitinib, 9 months for cabozantinib, 2.8 months for crizotinib, and 3 months for savolitinib. The confirmed overall response rate was 4% in patients treated with sunitinib, 23% with cabozantinib, 0% with crizotinib, and 3% with savolitinib, while the median overall survival was 16.4 months in patients treated with sunitinib, 20 months in the cabozantinib cohort, 19.9 months in the crizotinib cohort, and 11.7 months with savolitinib [175]. Cabozantinib in monotherapy showed important benefits in patients with pRCC as well, showing a complete response in some cases, while no similar result was described when compared to sunitinib, savolitinib, and crizotinib. Significantly higher survival rates with cabozantinib were observed when compared with other systemic agents like sunitinib, savolitinib, and crizotinib (20.0 months versus 16.4, 11.7, and 19.9 months) [175]. The phase IIIb/IV CheckMate 374 study aimed to assess the effectiveness of nivolumab in monotherapy in nccRCC individuals. The article is unique owing to the inclusion of BM RCC patients and suggests that a lot of promising results were noticed in the use of nivolumab as a single agent (dose of 240 mg), concluding encouraging effectiveness in these patients, with an OS rate of 16.3 months [176], in comparison to sunitinib (16.2 months) or everolimus (14.9 months) [177].

## 9. Multimodal Approach

Many systemic agents and cell-based vaccines showed potential benefits in the treatment of RCC and mRCC [87]. However, after a few years of treatment, a significant number of patients acquire resistance. In ccRCC, anti-PD-1 antibodies showed a superior response to TKIs, yet the ORR is still only up to 58% [28]. Multimodal approaches including radiation therapy, radical nephrectomy, and TKI revealed good outcomes in patients with BM RCC, with significant local control [178,179,180,181]. 

In individuals who are unfit or inoperable, the technique of renal arterial embolization (RAE) may be beneficial. Cases of complete tumoral devascularization, alleviation of pain, and visible hematuria have been reported [152]. 

### 9.1. Combination of Systemic Agents

Potential first-line options for patients with mRCC were nivolumab plus cabozantinib (in every IMDC risk), nivolumab/ipilimumab [182,183], cabozantinib/atezolizumab [184]. The therapeutic combination of nivolumab and cabozantinib not only reduces the risk of death by 30% but also leads to a complete therapeutic response in some individuals with mRCC. These findings were specifically noteworthy when the aforementioned systemic agents were compared to sunitinib alone (complete response rates of 12% for combination versus 5% for sunitinib monotherapy, and median PFS of 16.6 months versus 8.3 months) [171]. As previously mentioned, the combination of nivolumab and cabozantinib is more effective than sunitinib alone in mRCC cases, especially when it comes to survival rates (PFS of 16.6 months versus 8.3) [185]. In patients with ccRCC, the CheckMate 914 phase III clinical trial demonstrated a lack of benefit with nivolumab plus ipilimumab when compared to placebo [186]. A median OS of 21.2 months in treatment-naive patients with mRCC has been reported after the administration of nivolumab plus ipilimumab, followed by nivolumab monotherapy [126]. In M1 no evidence of disease (after resection of oligometastatic sites ≤1 year from nephrectomy) with ccRCC, post-surgical adjuvant pembrolizumab is inadvisable [187]. When compared to sunitinib alone, the therapeutic combination of lenvatinib and pembrolizumab showed superior results in individuals with mRCC (median PFS 9 months versus 24), while the therapeutic combination of lenvatinib and everolimus concluded almost similar survival rates [188,189]. In mRCC cases, temsirolimus in monotherapy has proven its efficacy in poor-risk ccRCC cases and should be considered a standard, while a future benefit in other types or subgroups of RCC may be demonstrated [190]. In patients with metastatic ccRCC who have never been treated before, the therapeutic combination of axitinib plus pembrolizumab has been proven superior to sunitinib alone, as demonstrated by the KEYNOTE-426 study (for the therapeutic combination, a median OS of 45.7 months has been reported in contrast with 40.1 months for the monotherapy) [191]. A significantly longer median PFS with avelumab plus axitinib compared with sunitinib, among patients who received these agents as first-line treatment for advanced RCC was concluded in comparison with sunitinib (8.4 months median PFS), while avelumab combined with axitinib showed superior efficacy (13.8 months median PFS) [192].

Telaglenastat is a micromolecule that selectively inhibits glutaminase and has an oral administration. Currently, telaglenastat (CB-830) is under clinical investigation for the treatment of various cancers, including RCC [193]. Telaglenastat did not improve the efficacy of cabozantinib in the mRCC (CANTATA trial), concluding a 9.2 month median progression-free survival (PFS) in the telaglenastat plus cabozantinib group in comparison to 9.3 months for the placebo plus cabozantinib group [194]. Another recent study evaluated telaglenastat plus cabozantinib (TelaC) or telaglenastat plus everolimus (TelaE) in mRCC. In the TelaC group, the median PFS was 6.2 months in patients with ccRCC and 7.7 months in patients with pRCC, while in the TelaE group, the median PFS for ccRCC was 5.5 months and for pRCC was 8.5 months [195]. The efficacy of everolimus combined either with telaglenastat (TelaE) or with placebo (PboE) in individuals with advanced/mRCC has been assessed (ENTRATA trial) (median PFS in the TelaE group has been 3.8 months in comparison to 1.9 months in the PboE group), encouraging the combination of glutaminase inhibition with mTOR inhibition [196]. 

The effectiveness of MK-6482 (belzutifan) has been addressed and is still being studied as monotherapy or combination therapy. MK-6482 has been initially developed as a curative option for the treatment of solid malignant tumors, including ccRCC and VHL disease-associated RCC [197,198]. The LITESPARK-024 study aims to evaluate MK-6482 alone or combined with palbociclib in individuals with ccRCC and is currently recruiting, with an estimated completion date of 16 March 2027 [173].

Several studies are currently addressing the matter of systemic therapy in patients with nccRCC. The administration of cabozantinib combined with atezolizumab in patients with advanced ccRCC and nccRCC (COSMIC-021 study) concluded with encouraging results. In ccRCC patients, the overall response rate was up to 58%, disease-control rates were up to 94%, and median progression-free survival was up to 19.5 months. In nccRCC individuals, the overall response rate was 31%, with a disease control rate of 94%, a median progression-free survival of 9.5 months, and a 39% rate of progression-free survival at one year [199]. Favorable therapeutic effects were noticed in most of the nccRCC subtypes with the cabozantinib plus nivolumab combination. These results were specifically noted in cases with papillary features. On the contrary, insufficient benefits were noted in chRCC [77]. The combination of systemic agents like savolitinib (a mesenchymal epithelial transition receptor inhibitor) and durvalumab (PD-L1 inhibitor) in 41 individuals with metastatic pRCC demonstrated a median progression-free survival of 4.9 months and 12.0 months in MET-driven patients, a median overall survival of 14.1 months in the treated population, and 27.4 months in MET-driven patients (CALYPSO RCT) [111]. Pembrolizumab in combination with lenvatinib as first-line therapy in patients with advanced nccRCC has been the main focus of the KEYNOTE-B61 phase II clinical trial. The trial is active but not recruiting anymore, with an estimated study completion date of October 22, 2025. To date, 49% of patients have shown a confirmed objective response, including 6% with a complete response and 44% with a partial response [200]. To date, there are no published phase III RCTs, including the nccRCC. However, STELLAR-304 [201] is a RCT that has a still-recruiting status and aims to evaluate in the future the efficacy of the combination of a next-generation TKI, zanzalintinib (generic name XL092) and nivolumab versus sunitinib, in patients with nccRCC. The anticipated study completion date is 1 June 2028 [202].

**Figure 2:**
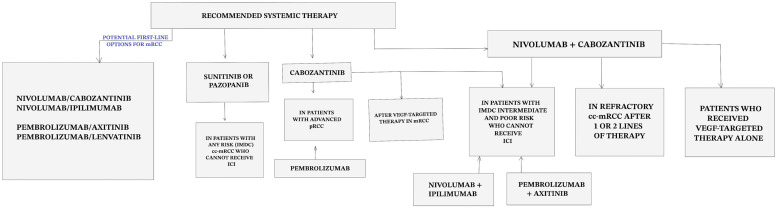
Summary of recommended systemic therapy in mRCC.

Currently, various options for first-line treatment have been proposed (Figure 2). Since there is no established consensus regarding the choice of systemic agents in mRCC, the medical community is still debating over different choices, as many therapeutic options have analogous efficacy. To date, there are no clear recommendations as to which is most appropriate for each patient [203]. Although most of the aforementioned adjuvant trials rely on clinical and histological tools for prognostic classification, the unique molecular and genomic signatures of patients have a complex effect on disease progression and treatment response and should rather be addressed for individualized care and better outcomes in patients with RCC [18,77,204,205,206,207]. 

### 9.2. Radiotherapy

Notwithstanding the controversial role of radiotherapy in mRCC, stereotactic radiosurgery (SRS) has been associated with good outcomes in a multimodal therapeutic approach [150] or when trying to defer systemic therapy in patients with mRCC [208]. The rationale behind the combination of immunotherapies and radiation therapy (SRS and whole brain radiotherapy—WBRT) was initially derived from abscopal effect observations [59]. Some systemic agents may help sensitize the tumor to radiation, for example, sorafenib (through inhibition of radiation-induced VEGFR2 and its downstream extracellular-signal-regulated kinase (ERK) signaling pathway) induces deoxyribonucleic acid (DNA) damage, and block/delay DNA repair. Sunitinib and temsirolimus have also been shown to increase radiosensitivity [209]. For multiple metastases, whole-body WBRT and SRS have been reported to improve OS [210,211,212]. Even though WBRT can be used in patients with more than 10 BM RCC (with hippocampal avoidance) [213], this method has a limited indication given its neurotoxicity. Because of this reason and the resistance of RCC to conventional fractionation radiotherapy, SRS is becoming more preferred even in patients with more BM RCC, especially when combined with systemic therapy when SRS can improve OS, local tumor control, and clinical symptoms [53]. Although the exact role and combination of WBRT and SRS in the treatment of BM RCC remain unclear, it has been demonstrated that the addition of SRS to WBRT improves survival and local and intracranial control in patients with one to three BM RCC (OS rates between SRS and SRS + WBRT: 12 vs. 16 months). WBRT alone must be reserved for patients with multiple metastases and a poor prognosis [214].

### 9.3. Neurosurgical Excision

Neurosurgical intervention is regarded as a gold standard approach in patients with single-symptomatic BM RCC, while surgical excision of the primary tumor was shown to be curative in the early stages of ccRCC, and survival rates exceed 50%. However, approximately one-third of patients with localized ccRCC relapse after surgical resection [213,215].

### 9.4. Cytoreductive Nephrectomy

As a significant part of the multimodal therapy of mRCC, cytoreductive nephrectomy (CN) is defined as the excision of the primary lesion in the presence of metastases [216,217]. In patients with mRCC, an OS benefit was found when treated with cytoreductive nephrectomy (CN) in comparison with individuals with no CN: median OS 42.4 months vs. 16.8 months in patients with good to intermediate-risk (according to the Memorial Sloan Kettering Cancer Center criteria) [218], as well as 40.0 months vs. 23.2 months [219], or 29.9 months vs. 18.1 months [220]. For intermediate-risk patients, OS in individuals with deferred CN was longer as opposed to patients with upfront CN (33 months versus 22) [221]. The Cancer du Rein Metastatique Nephrectomie et Antiangiogéniques (CARMENA) trial showed that sunitinib in monotherapy was not inferior to sunitinib associated with CN in individuals with metastatic ccRCC. Median OS was longer in the sunitinib monotherapy group than in the sunitinib-nephrectomy group (23.4 vs. 19.0 months and 13.3 vs. 10.2 months for the intermediate-risk, and poor-risk group, respectively) [222]. Therefore, in patients with intermediate-risk RCC, deferred CN might be a suitable treatment option [223]. However, when it comes to the benefits of CN in patients with mRCC, debates and controversy still exist, as CN remains a moving target [217,224,225].

## 10. Adverse Reactions and Toxicity Associated with Systemic Therapy

Although systemic therapy has been proven beneficial in many solid tumor malignancies, it can often be correlated with wide-ranging side effects (immune-related adverse events, irAEs). In a variety of cancer pathologies, immunotherapy is used either alone or in combination with other systemic agents or therapies, which may result in potential toxic effects that can be life-threatening and require immediate awareness and management [94]. Multiple attempts to counter resistance mechanisms have been a subject of study, yet unfortunately, no current successful solution to this problem has been described. Some studies approached a new subject regarding HIF2α inhibitors (e.g., MK-6482—belzutifan) and the matter of targeting the HIF2–VEGF axis, but the subject is still a field of active development and research [226,227,228]. The most frequent side effects associated with systemic agents are summarized in Table 3. 

## 11. Conclusions

Despite the fact that patients with metastatic RCC, specifically BM RCC, are reported to have poor outcomes and low OS rates, the emergence of systemic targeted agents has been considered beneficial in these categories of population. Although many patients with BM RCC have been considered ineligible for clinical trials, new studies approach the issue, suggesting widening the spectrum of inclusion criteria as many new researchers conclude the efficacy of systemic agents in the multimodal therapeutic approach of BM RCC. Various studies research the blocking of the proliferation of cancerous cells and the inhibition of angiogenesis. The best results in real-world patients with BM RCC were obtained with the multimodal therapeutic approach. Despite many serious hindrances that still exist and cures that remain unusual, BM RCC *molecular pathways*, diagnosis, and therapy represent an attractive field. Hopefully, novel technologies will contribute more in order to uncover new opportunities for this pathology and to improve the currently poor prognosis, as this remains the most arduous task regarding patients with mRCC.

## Figures and Tables

**Table 1:** Summary of available systemic therapeutic agents for mRCC and BM RCC.

*Classification and Category*	*Agent Name*	*Mechanism of Action*	*Central Nervous System Penetration*
**I.** **Immune Checkpoint Inhibitors** [40,53,77]	Nivolumab, Pembrolizumab [40]; Atezolizumab [40]; Avelumab [53] Durvalumab [77], Ipilimumab [53]	Anti-programmed cell death ligand 1 (PD-L1) [40,53] Anti-cytotoxic T lymphocye-associated antigen 4 (CTLA-4) [53]; Blocking the binding of PD-L1 to PD-1 and cluster of differentiation 80 [77]; Anti-cytotoxic T lymphocye-associated antigen 4 (CTLA-4) [40,53]	It is still not clear whether this category of systemic agents can travel across the BBB; however, animal studies show moderate penetrance, while many clinical studies have demonstrated intracranial response [67,78,79].
**II.** **Receptor Tyrosine kinase inhibitors and monoclonal antibodies against circulating VEGF** [40,53,80,81]	Sunitinib, Cabozantinib, Axitinib, and Lenvatinib [53]; Sorafenib [81]; Erlotinib, Bevacizumab [40]	Inhibition of tyrosine kinase activity or tumor anti-angiogenesis [80]	This category of systemic agents has a lower molecular weight and good BBB permeability [34,35,82,83,84,85,86,87].
**III.** **Mammalian target of rapamycin inhibitors** [53,88,89,90]	Everolimus [53,88], Temsirolimus [40]	Stimulates the degradation of cycline D1 and is involved in the phospho-p70 S6 kinase downregulation [89,90]	mTOR inhibitors are lipophilic agents with good BBB penetration in animal models and anti-tumor activity in real-life patients [75,91,92,93].
**IV.** **Chemotherapy (platinum-based regimens/pyrimidine analog) ^1^** [40,94]	5-fluorouracil [95]; Anthracyclines, Gemcitabine, Cisplatin, Carboplatin ^☨^, and Paclitaxel [40]	Inhibits thymidylate synthase and/or the replication of the DNA [95,96,97]; induction of carboplatin-DNA adducts; anti-depolymerization of microtubules; and cell cycle arrest at the G2/M phase [98]	Due to BBB disruption in BM, the penetrance of chemotherapy is not limited, but the administration should be based on the chemosensitivity of the primary tumor [99].

^1^ The role of chemotherapy is limited to the use of platinum-based chemotherapy in patients with non-clear-cell collecting duct and renal medullary carcinomas [40,100]. ^☨^ Pulsed ultrasound showed blood–brain barrier disruption in a clinical trial and optimized the delivery of chemotherapy to the brain in certain brain tumors [101].

**Table 2:** Proposed systemic therapy for different types of metastatic RCC.

*Pathology*	*Proposed Systemic Therapy*
**Clear-cell RCC** **^◆^**	**AMERICAN SOCIETY OF CLINICAL ONCOLOGY (ASCO) GUIDELINES [52]** **FIRTS LINE:** Combinations of ICIs (i.e., Ipilimumab, Nivolumab) or ICI + VEGFR TKI; VEGFR monotherapy or ICI monotherapy in selected patients (e.g., co-existing medical problems); **SECOND OR LATER LINE** Nivolumab or Cabozantinib ^✢^ VEGFR TKI (for progression on combination of ICI (e.g., Nivolumab, Ipilimumab), and for patients who progress on ICI + VEGFR TKI. **EUROPEAN SOCIETY OF MEDICAL ONCOLOGY (ESMO) GUIDELINES [102]** **FIRST LINE** PD-1 inhibitor therapy + VEGFR-targeted therapy; PD-1 inhibitor + CTLA-4 inhibition (e.g., Lenvatinib–Pembrolizumab, Axitinib–Pembrolizumab, Cabozantinib–Nivolumab, Ipilimumab–Nivolumab; **If immunotherapy is contraindicated/not available:** Pazopanib, Sunitinib, Tivozanib; Cabozantinib—in patients who cannot receive first-line PD-1 inhibitor-based therapy; **SECOND LINE** A VEGFR systemic agent that has not been previously given (Axitinib, Cabozantinib, Pazopanib, Sunitinib) or Lenvatinib–Everolimus. **EUROPEAN ASSOCIATION OF UROLOGY (EAU) GUIDELINES [103]** **FIRST LINE** ICIs targeting PD-1, complemented by a TKI, or a second ICI directed against CTLA-4 (Nivolumab–Cabozantinib, Pembrolizumab–Axitinib, Pembrolizumab–Lenvatinib, Nivolumab–Ipilimumab) **For patients who cannot receive ICI:** Pazopanib, Cabozantinib, Sunitinib; **SECOND OR LATER LINE** In patients with prior immunotherapy: Anti-VEGF that has not been previously administered + Immunotherapy; In patients with prior TKI: Nivolumab, Cabozantinib. Alternative: Axitinib.
**Papillary RCC**	**ESMO GUIDELINES [102]** **FIRST LINE** Cabozantinib Alternative: Sunitinib, Pembrolizumab, Savolitinib in MET-altered tumors; **SECOND LINE** A systemic agent that has not been given previously to the aforementioned agents. **EAU GUIDELINES [103]** Cabozantinib; **OTHER STUDIES** MET-inhibitor-based approach for MET-driven papillary tumors (Crizotinib, Foretinib, Savolitinib) *, Pazopanib **, Axitinib ** [27,40,104,105].
**Chromophobe RCC**	**EAU GUIDELINES [103]** Sunitinib (based on results from the phase II ASPEN clinical trial) [106]; **OTHER STUDIES** Everolimus [106,107], Sunitinib [108], Levantinib–Everolimus [109,110], Bevacizumab–Everolimus [111], Nivolumab–Cabozantinib *** [77], Bevacizumab–Atezolizumab *** [112].
**Collecting duct and renal medullary carcinoma**	**OTHER STUDIES** **Cytotoxic chemotherapy is suggested as first approach** (Cisplatin–Gemcitabine, Carboplatin–Gemcitabine, Carboplatin–Paclitaxel) [113,114,115,116,117]; Anthracyclines ****, Bortezomib ****, Sunitinib **** [118,119,120,121].
**Trans-locational RCC**	**OTHER STUDIES** Sunitinib, Nivolumab, Cytokine therapy (interferon-α or interleukin-2) [122,123]; Atezolizumab, Ipilimumab [124].
**Unclassified RCC**	**EAU GUIDELINES [103]** Sunitinib (based on results from the phase II ASPEN clinical trial) [106]; **OTHER STUDIES** Nivolumab–Ipilimumab [125,126,127]; Pembrolizumab [128]; Sunitinib monotherapy [106,129]; Cabozantinib monotherapy [130]; Levantinib–Everolimus [131]; Bevacizumab + Everolimus [132].
**Sarcomatoid RCC**	**ASCO GUIDELINES [52]** **FIRST LINE** Combination of ICIs, or alternatively ICI + VEGFR TKI **ESMO GUIDELINES [102]** **FIRST LINE** ICI

* Still evolving (experimental agents). ** Less preferred therapeutic agents. *** Limited efficacy concluded in chRCC. **** Limited activity in this pathology. ^✢^ If progressed on a VEGFR TKI alone [52]. ^◆^ American Society of Clinical Oncology Guidelines on Management of Metastatic Clear-Cell Renal Cell Carcinoma (ASCO Guideline, 2022) have no optimal systemic recommendation for BM RCC [52].

**Table 3:** Common and less common side effects of targeted agents administered in patients with mRCC and BM RCC.

*Systemic Adverse Events*	*Types of Events*	*Systemic Agents Associated with irAEs*
**Dermatological reactions**	Inflammatory skin reaction [126,128,229,230,231], Immunobullous disease, Vasculitis, Neutrophilic dermatoses, Hand–foot syndrome (HFS) [190,232]	Nivolumab–Ipilimumab [126,229], Bevacizumab + IFN-α [232], Sorafenib [232], Sunitinib [232], Pazopanib, Axitinib [232], Sunitinib [190], Sorafenib [190], Temsirolimus [190], Combination of ICIs [231], Pembrolizumab [128], Nivolumab [230]
**Gastrointestinal**	Constipation, Diarrhea [59,94,126,128,153,190,199,229,230,232,233], Mucositis [232,233], Colitis [59,94,126,128,153,229,233], Hepatotoxicity [94,126,229,230], Emesis [59,126,232,233], Anorexia [232], Dysgeusia [199,232,233]	Nivolumab–Ipilimumab [126,229], Nivolumab [230], Bevacizumab + IFN-α [232], Sorafenib [232], Sunitinib [232], Pazopanib, Axitinib [232], Sunitinib [190], Cabozantinib–Nivolumab [233], Cabozantinib–Atezolizumab [199], Pembrolizumab [128,153], Combination of ICIs [59,94,231]
**Respiratory**	Dyspnea, Pneumonitis (may be delayed) [59,94,126,128,153,230,233], Cough [128,231]	Nivolumab–Ipilimumab [126], Nivolumab [230], Cabozantinib–Nivolumab [233], Pembrolizumab [128,153], Combination of ICIs [59,94,231]
**Endocrinopathies and Metabolic disturbances**	Hashimoto disease [59,126,153], Graves’ disease, Hypothyroidism [94,126,128,153,199,230,231,232,233], Hyperthyroidism [94,126,153,233], Hypophysitis [59,94,126,153,229,233], Adrenal insufficiency [94,126,128,153,233], Hyperlipidemia, Hypophosphatemia [232], Type 1 diabetes mellitus [94,128,153], Increased serum glucose [230]	Nivolumab–Ipilimumab [126,229], Nivolumab [230], Bevacizumab + IFN-α [232], Sorafenib [232], Sunitinib [232], Pazopanib, Axitinib [232], Cabozantinib–Nivolumab [233], Cabozantinib–Atezolizumab [199], Pembrolizumab [128,153], Combination of ICIs [59,94,231]
**Opportunistic infections**	Rare, unusual (e.g., Pneumocystis pneumonia) [94,234]	ICIs [94,234]
**Neurologic**	Headache [59,94,128], Peripheral sensory neuropathy [94,231], Guillain–Barré syndrome [94,231], Cranial neuropathies [153,230,231], Myasthenia gravis [229], Encephalopathy [59,231], Aseptic meningitis [231], Enteric neuropathy, Transverse myelitis, Pancerebellitis, Autoimmune encephalitis; Insomnia, Myastenia Gravis [231], Ptosis [231]	Nivolumab [230], Nivolumab–Ipilimumab [229], Pembrolizumab [128,153], Combination of ICIs [59,94,231]
**Cardiovascular**	Hypertension [190,200,230,231,232,233] Myocarditis [94], Myocardial infarction [153], Pericarditis [94], Heart failure [94], Arrhythmias [94,153], Vasculitis [94], Venous thromboembolism [94]	Nivolumab [230], Bevacizumab + IFN-α [232], Sorafenib [232], Sunitinib [232], Pazopanib, Axitinib [232], Sorafenib [190], Bevacizumab [190], Cabozantinib–Nivolumab [233], Pembrolizumab [153], Pembrolizumab–Lenvatinib [200], ICIs [94,231]
**Hematologic**	Thrombocytopenia [94,153,190,232], Acquired red cell aplasia, Acquired hemophilia A [94], Autoimmune hemolytic anemia [94], Hemophagocytic syndrome [94], Neutropenia [94,128,153,190,232], Cryoglobulinemia [94]	Bevacizumab + IFN-α [232], Sorafenib [232], Sunitinib [232], Pazopanib, Axitinib [232], Sunitinib [190], Pembrolizumab [94,128,153], ICIs [94]
**Rheumatologic and musculoskeletal**	Muscular weakness [126], Inflammatory arthritis, Sicca syndrome, Vasculitis [94], Arthralgia [128,153]	Nivolumab–Ipilimumab [126], Pembrolizumab [128,153], ICIs [94]
**Less frequent side effects**	Eye: Episcleritis, Conjunctivitis, Uveitis, Orbital inflammation [94]; Kidney: Acute kidney injury [94,153], Proteinuria [199,200], Increased Serum creatinine [126,231,233]; Exocrine pancreas dysfunction [94], Tremor [229,230], Pyrexia [126,128,153], Fatigue [128,190]	Nivolumab–Ipilimumab [126,229], Nivolumab [230], Sunitinib [190], Sorafenib, Cabozantinib–Nivolumab [233], Cabozantinib–Atezolizumab [199], Pembrolizumab [128,153], Combination of ICIs [94,231], Pembrolizumab–Lenvatinib [200]

## Data Availability

Not applicable.
